# Bad Blood: An Unusual Case of Small Bowel Obstruction From Hematoma-Induced Intussusception

**DOI:** 10.7759/cureus.106844

**Published:** 2026-04-11

**Authors:** Gustavo Christian-Colón, Derick Rodriguez-Reyes, Arnaldo Figueroa-Tejada, Gabriel Figueroa-Martínez, Emmanuel Belardo-Del Rio, Adel Gonzalez-Montalvo

**Affiliations:** 1 General Surgery, Hospital Episcopal San Lucas, Ponce, PRI; 2 General Surgery, University of Puerto Rico, Medical Sciences Campus, San Juan, PRI

**Keywords:** adult small bowel intussusception, anticoagulation-associated bleeding, hematoma-induced intussusception, ileo-ileal intussusception, intraluminal hematoma, intramural small bowel hematoma, small bowel intussusception, small bowel obstruction (sbo), spontaneous gastrointestinal hematoma

## Abstract

Small bowel obstruction (SBO) is a frequent surgical emergency with a broad differential diagnosis, ranging from adhesions and malignancy to rare causes such as intraluminal blood clots and spontaneous intramural hematomas. These uncommon etiologies are often associated with anticoagulation therapy and can present diagnostic and therapeutic challenges.

We report the case of a 73-year-old male with a history of abdominal aortic aneurysm (AAA) and long-term antihypertensive therapy, presenting with a three-day history of diffuse abdominal pain and persistent vomiting. Imaging revealed small bowel intussusception with associated hematoma, raising concern for a neoplastic lead point. Emergent laparotomy confirmed a mid-ileal intussusception and an intraluminal hematoma, prompting segmental small bowel resection. The postoperative course was complicated by new-onset atrial fibrillation, septic shock, pancytopenia, and acute kidney injury, requiring intensive multidisciplinary management. A second-look laparotomy identified a hematoma at the anastomotic site, which was evacuated successfully.

This case illustrates a rare cause of SBO hematoma-induced intussusception, potentially related to vascular comorbidities. Early surgical intervention based on clinical suspicion and radiographic findings was essential. The complexity of the patient’s postoperative course underscores the importance of coordinated multidisciplinary care in managing similar high-risk patients.

Uncommon etiologies such as intraluminal and intramural hematomas should be considered in the differential diagnosis of SBO, particularly in patients on anticoagulation or with significant vascular disease. Timely diagnosis and appropriate surgical management are critical for favorable outcomes. This case contributes to the limited literature on hematoma-induced intussusception and reinforces the importance of vigilant postoperative monitoring.

## Introduction

Small bowel obstruction (SBO) is a significant clinical entity, accounting for up to 16% of hospital admissions for abdominal pain and associated with a mortality rate ranging from 2% to 8% [1]. This figure may rise to 25% in cases complicated by bowel ischemia and subsequent perforation [1]. Clinically, SBO typically presents with crampy abdominal pain, abdominal distension, and persistent emesis [2].

The etiologies of SBO are diverse. Adhesions are the most common cause, responsible for approximately 70% of cases [1,2]. Other etiologies include hernias, malignancies, Crohn’s disease, volvulus, gallstone ileus, and foreign body obstructions [1,2]. In patients without prior abdominal surgery, a so-called “virgin abdomen" SBO may result from malignancy, internal hernias, or bezoars [3]. Notably, the World Society of Emergency Surgery reports that adhesions can still contribute to obstruction in this population, emphasizing the complex and multifactorial nature of SBO pathogenesis [3].

Intussusception, while a well-recognized cause of bowel obstruction in children, is rare in adults, accounting for only 1% to 5% of cases [4]. When it occurs, it is usually secondary to a pathological lead point such as neoplasia, inflammatory bowel disease, or postoperative adhesions [4]. Intussusception involves the telescoping of a proximal bowel segment into an adjacent distal segment, which can lead to obstruction and, if untreated, ischemia [4]. Computed tomography (CT) remains the most sensitive modality for identifying intussusception and for distinguishing cases with a lead point from transient, self-limiting variants [4]. Thus, imaging plays a critical role in guiding surgical versus conservative management.

Although SBO is a common surgical emergency, adult intussusception remains a rare but important cause. Prompt recognition through appropriate imaging and a high index of suspicion are essential to guide management. In this report, we present a case of adult SBO due to intussusception caused by an intraluminal hematoma, which is an unusual and rarely documented pathologic lead point.

## Case presentation

A 73-year-old male presented to the emergency department with a three-day history of persistent abdominal pain and vomiting. One week prior, he had visited an outside hospital for similar symptoms, where imaging was obtained, intravenous hydration was administered, and he was subsequently discharged. Due to ongoing symptoms and lack of clinical improvement, he sought further evaluation.

He described the abdominal pain as intermittent but severe when present, sharp, diffuse, non-radiating, and without identifiable relieving factors. The episodes were accompanied by non-bloody, non-bilious vomiting. Patient denied fever, diarrhea, or hematemesis. His last colonoscopy, performed two years prior, was unremarkable.

His past medical history included hypertension, a descending thoracic aortic aneurysm, and an abdominal aortic aneurysm (AAA) with dissection, all managed medically with blood pressure control. Home medications included lisinopril, nifedipine, atorvastatin, and ferrous sulfate. Patient denied anticoagulation use at home. He reported an allergy to aspirin and had no prior intra-abdominal surgical history. He was a former smoker with a half-pack-per-day habit over several years.

On presentation, vital signs were notable for stable blood pressure and afebrile status. He was tachycardic with a regular rhythm at initial evaluation. The patient was alert, oriented, and in no acute distress but appeared frail and malnourished. Chest examination revealed symmetric expansion and unlabored respirations on room air. Abdominal examination showed mild distension, high-pitched borborygmi on auscultation, and a tympanic, soft, and depressible abdomen. He had diffuse abdominal tenderness without rebound, guarding, rigidity, or other signs of peritonitis.

Initial laboratory evaluation demonstrated stable hemoglobin and no leukocytosis. The chemistry panel was notable for elevated lactic acid, elevated creatinine, and metabolic alkalosis, consistent with volume depletion from ongoing emesis. The patient was made nil per os (NPO), and aggressive intravenous fluid resuscitation was initiated. A nasogastric tube (NGT) was placed, yielding approximately two liters of feculent output, resulting in mild symptomatic relief.

**Figure 1:**
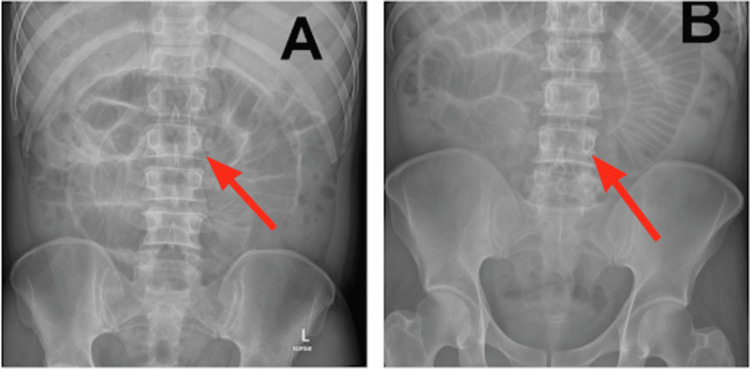
Upright abdominal X-ray A, B: dilated bowel loops with plicae circulares, consistent with small bowel obstruction. Red arrows highlight plicae circulares, consistent with dilated small bowel.

**Figure 2:**
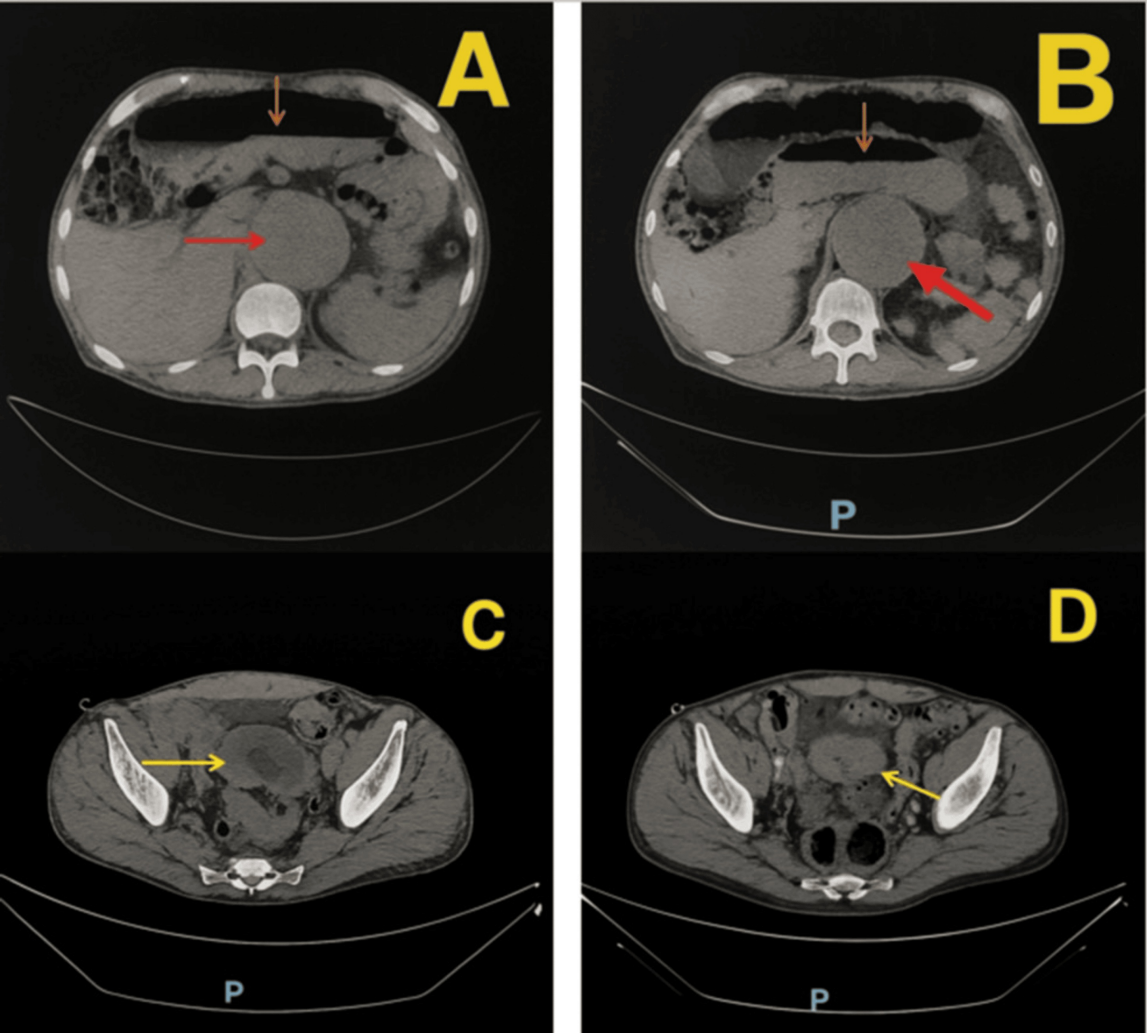
Abdominopelvic CT findings A: abdominal aortic aneurysm (red arrow), with dilated proximal duodenum with air-fluid levels (orange arrow); B: dilated distal duodenum and proximal jejunum with air-fluid levels (orange arrow) and an abdominal aortic aneurysm (red arrow); C: distal ileal-ileal small bowel intussusception with classic “target sign" appearance (yellow arrow); D: bowel narrowing distal to intussusception with hyperintensity (yellow arrow).

Given his history of thoracic and AAAs, a vascular surgery consult was obtained. No acute vascular intervention was deemed necessary, as the aneurysms were diagnosed eight years prior and interval imaging had remained stable. Abdominal radiography revealed dilated bowel loops with visible plicae circulares, consistent with a potential SBO. CT of the abdomen without intravenous contrast demonstrated distal small bowel intussusception and marked dilation of both the proximal and distal duodenum. Abdominal radiography and abdominopelvic CT scans are shown in Figures 1, 2. 

Abdominal CT imaging revealed air-fluid levels throughout the stomach, duodenum, and jejunum. In the mid-ileum, the classic "target sign" was observed, consistent with intussusception, along with segmental narrowing distal to the involved bowel with luminal hyperintensity. These findings raised concern for a pathological lead point, with neoplasm considered in the differential diagnosis.

Given the radiologic evidence of mechanical obstruction with suspected intussusception, the potential for an underlying malignancy, and the patient’s lack of clinical improvement despite nasogastric decompression and supportive care, an emergent exploratory laparotomy was warranted with subsequent small bowel resection. The decision to proceed surgically was made to relieve the obstruction, identify the lead point, and prevent progression of the small intestine to subsequent bowel ischemia or perforation.

In the operating room, after the patient was placed in the supine position, general anesthesia was induced, prophylactic antibiotics were administered, and the abdomen was prepped and draped in the usual sterile fashion. A midline laparotomy was performed, and the abdominal wall was dissected down to the peritoneal cavity. Upon entry, a large volume of peritoneal fluid was encountered and sent for cytopathological analysis.

Inspection of the liver and peritoneal surfaces revealed no evidence of metastatic disease. The small bowel appeared diffusely distended and was carefully exteriorized. An intussusception was identified in the mid-ileum. After establishing appropriate proximal and distal margins, a segmental small bowel resection was performed using an Endo GIA 60 mm stapler (Medtronic plc, Dublin, Ireland) with a yellow cartridge. The mesentery was divided using a bipolar energy device (LigaSure™, Medtronic plc, Dublin, Ireland), and the enterotomy was closed with a TA stapler (Medtronic plc, Dublin, Ireland). The mesenteric rent was then closed using 3-0 Vicryl sutures.

A thorough inspection of the bowel from the ligament of Treitz to the rectum was performed. Patchy areas of mucosal congestion were observed in the proximal jejunum, raising concern for possible embolic phenomena, although there were no signs of frank ischemia. Given the concern for evolving ischemia and the patient's overall condition, a decision was made intraoperatively to defer a primary anastomosis and perform a temporary abdominal closure using a Bogotá bag. The bag was secured to the skin with 2-0 Prolene sutures to facilitate a planned second-look laparotomy.

The patient tolerated the procedure without intraoperative complications. However, he developed hypotension and tachycardia with an irregular rhythm at the conclusion of the operation. Electrocardiography confirmed new-onset atrial fibrillation. The patient was transferred to the recovery unit, intubated, on mechanical ventilation, and started on vasopressor and amiodarone infusions. He was subsequently admitted to the intensive care unit for further management.

Following the development of new-onset atrial fibrillation, internal medicine was consulted. Given that the patient had converted to sinus rhythm during their evaluation, the amiodarone infusion was discontinued. Nephrology was also consulted for the management of acute kidney injury. Their recommendations included continued intravenous fluid resuscitation and maintenance of vasopressor support.

Postoperative laboratory workup revealed leukopenia, thrombocytopenia, anemia, and a serum creatinine level of 1.69 mg/dL, consistent with acute kidney injury. Septic shock was suspected as the etiology of the patient’s intraoperative hypotension. Consequently, a sepsis bundle was initiated, and the patient remained in the intensive care unit (ICU) for further monitoring and management. 

Due to the risk of hemorrhage, anticoagulation was not administered, and the patient was placed on sequential compression stockings for thromboembolism prophylaxis for the remainder of hospitalization. On postoperative day one, the patient was taken back to the operating room for a planned re-exploration. After induction of anesthesia and sterile preparation, the Bogotá bag was removed. Intraoperatively, a moderately sized hematoma was identified adherent to the anastomotic line. The hematoma was carefully evacuated, revealing mild arterial bleeding at the staple line. Hemostasis was achieved using multiple 3-0 Vicryl figure-of-eight sutures. Special care was taken to preserve luminal patency during repair. The small bowel remained congested but showed no signs of frank ischemia. The abdominal cavity was irrigated with sterile saline, and hemostasis was confirmed. The Bogotá bag was reapplied and secured to the skin with 2-0 Prolene sutures. Sterile dressings were placed, and no intraoperative complications occurred. The patient was returned to the ICU in stable condition.

While in the ICU, the patient was found to be pancytopenic and required an additional transfusion of packed red blood cells (PRBCs). In total, he received four units of PRBCs following his initial laparotomy due to recurrent hemoglobin levels below 7.0 g/dL. He was successfully extubated two days after the second surgery and continued to improve clinically, with improved abdominal symptoms and tolerating soft diets that were eventually progressed to regular. The patient was subsequently transferred to the surgical ward and discharged home on postoperative day 12, and was scheduled to follow up in outpatient clinics.

Pathologic analysis of the resected specimen from the initial operation confirmed small bowel intussusception, with an intraluminal hematoma identified as the pathologic lead point. No evidence of neoplasia was observed. The SBO pathology report stated that the patient presented with a clinical history of high-grade SBO secondary to ileo-ileal intussusception, with both pre-operative and post-operative diagnoses consistent with this condition (as per the operative report). Pathologic evaluation of a 27 cm segment of ileum obtained via partial enterectomy confirmed intussusception associated with significant luminal narrowing and obstruction. Gross examination revealed a segment of ileum measuring 27 cm in length and 4.5 cm in diameter, with the intussuscepted area located 7 cm from the nearest margin. The serosal surface appeared smooth but congested. The bowel was diffusely dilated, except for a 2 cm segment narrowed to 3 cm in diameter, corresponding to the site of intussusception. Upon opening, the lumen contained blood clots, and the bowel wall was notably thin with a hemorrhagic mucosal surface, consistent with ischemic changes secondary to obstruction.

A separate cytopathology report corresponds to a specimen obtained from the peritoneal cavity via ascitic fluid paracentesis. The specimen was collected on December 16, 2024; received on December 23, 2024; and the final report was issued on December 30, 2024. Cytologic examination demonstrated reactive mesothelial cells. Immunohistochemical staining showed negativity for Ber-EP4 (Ep-CAM), MOC-31 (ERA), and synaptophysin, while calretinin and CK5/6 were positive, supporting a mesothelial origin of the cells (Table 1). Overall, the findings are consistent with reactive mesothelial proliferation, and the specimen was negative for malignancy.

**Table 1:** Cytopathology report from the peritoneal cavity showing mesothelial cells positive for calretinin and CK5/6

Field	Result
Specimen	Peritoneal cavity, ascitic fluid paracentesis
Brief Clinical Summary	Ileo-ileal intussusception
Diagnostic Impression	Reactive mesothelial cells
Immunohistochemistry	
Ber-EP4 (Ep-CAM)	Negative
Calretinin	Positive
CK5/6	Positive
(MOC-31) ERA	Negative
Synaptophysin	Negative
Diagnosis	Negative for malignancy

## Discussion

This case presents a rare etiology for bowel obstruction. Notably, cytopathologic analysis revealed mesothelial cells positive for calretinin and CK 5/6, which are linked with malignancy such as mesothelioma [5]. However, no signs of malignancy were found intraoperatively to suggest mesothelioma as the etiology behind the intussusception. In addition, to our knowledge, current literature provides no established association between these markers and the pathophysiology of hematoma formation or intussusception. Further investigation is needed to explore any potential clinical relevance of these findings in similar cases.

SBO caused by intussusception with an intraluminal hematoma serving as the lead point is exceedingly rare, particularly in the absence of anticoagulation or trauma. The current literature, comprising case reports and small series, demonstrates that intramural or intraluminal hematomas most often occur secondary to warfarin use, blunt abdominal trauma, or hematologic disorders such as hemophilia, and in these settings, they can function as pathological lead points for intussusception [6-11].

As per the limited previous case reports on this topic, adult cases of small bowel hematoma are associated with warfarin-induced coagulopathy, where the hematoma resulted in luminal narrowing and mechanical obstruction [7,8]. Similar to our case, these patients presented with obstructive symptoms such as abdominal pain, abdominal distension, and emesis. However, unlike our patient, anticoagulation was the primary underlying factor. Notably, in both reports, CT imaging demonstrated intramural hyperdensity and bowel wall thickening, hallmarks that were also observed in our patient's imaging, though in our case, the CT findings were most striking for the classic "target sign" of intussusception rather than diffuse mural thickening.

Additionally, it was reported that an adult female on warfarin developed a large ileal intramural hematoma leading to SBO [9]. Interestingly, the hematoma was also suspected intraoperatively as a potential neoplastic lesion, prompting bowel resection, a scenario similar to this one, where imaging raised concern for malignancy and surgical exploration was indicated due to clinical non-improvement and diagnostic uncertainty. Both cases highlight the diagnostic difficulty posed by such rare presentations and the necessity of operative intervention when imaging is inconclusive or symptoms persist despite fluid hydration, NPO, and nasogastric decompression management. 

Intraluminal hematomas leading to intussusception have been mainly described in pediatric populations [6]. The literature describes hematoma-induced intussusception and bowel obstruction in pediatric patients, namely in the setting of trauma or bleeding disorders [6,10]. Case series have found that, although rare, the intraluminal hematoma, regardless of cause, can serve as a physical lead point that initiates telescoping of the bowel. This serves as an explanation behind the mechanism of disease that led to distal small bowel telescoping and subsequent obstruction. 

This pathophysiologic mechanism is supported by a review of unusual radiological findings in childhood intussusception [11]. It was emphasized that while classic lead points are often pathological (e.g., malignancy, polyps, Meckel’s diverticulum), hematomas should remain in the differential diagnosis, especially when imaging reveals a hyperdense mass within the bowel wall or lumen, which mirrors the radiological characteristics seen in our patient [11].

What distinguishes our case from previous literature is the absence of previous anticoagulation or identifiable bleeding disorder within the context of an adult patient. Instead, the presence of thoracic and AAAs suggests an alternative vascular mechanism. This is possibly related to underlying vessel wall fragility and systemic arteriopathy that may present as gastrointestinal bleeding, such as aortoenteric fistula [12]. To our knowledge, there is no current evidence in the literature that aortic aneurysms, in the absence of anticoagulation or trauma, predispose patients to the development of small bowel intramural hematoma that may serve as a lead point for intussusception. Hence, the aforementioned hypothesis is not well-documented in the literature, but it raises an important consideration for future research and case reporting. The identification of an intraluminal hematoma as the lead point, confirmed on pathology, adds to the very limited body of evidence that such events may arise even without traditional risk factors.

In summary, while prior reports have linked hematoma-associated intussusception to trauma, warfarin therapy, or coagulopathies, our case is unique in its association with underlying vascular disease in the absence of these factors. This highlights a potentially underrecognized mechanism of hematoma formation in aneurysmal patients and expands the differential diagnosis for intussusception in adult individuals presenting with bowel obstruction.

## Conclusions

This case highlights a rare and diagnostically challenging presentation of SBO caused by intussusception with an intraluminal hematoma as the lead point in a patient without anticoagulation therapy or coagulopathy, but with known thoracic and AAAs. While intramural hematomas have been well described in association with anticoagulant use and trauma, the presence of an intraluminal hematoma leading to intussusception in the absence of such factors is exceptionally uncommon.

The key takeaways from this case report are the following: adult intussusception is rare and frequently associated with a pathologic lead point, most commonly neoplasms, but rarely, hematomas; intraluminal hematomas can act as lead points for intussusception, even in patients without anticoagulant use, particularly in those with vascular comorbidities such as aortic aneurysms; CT remains the most sensitive modality for detecting intussusception and may aid in identifying hematomas, though findings may mimic malignancy; and early surgical exploration should be considered when there is clinical deterioration or diagnostic uncertainty, especially in high-risk patients.

The findings emphasize the need for heightened clinical suspicion and timely operative intervention when dealing with unexplained bowel obstruction in patients with underlying vascular pathology. This case also expands the differential diagnosis for adult intussusception and underscores the importance of individualized intraoperative decision-making when encountering rare causes of obstruction.
